# In-Situ Ligand-Induced Chirality Transfer in Emissive CdSe Nanoplatelets

**DOI:** 10.1021/acs.jpclett.6c01325

**Published:** 2026-06-17

**Authors:** William Girten, Farwa Awan, Nikita S. Dutta, Marissa Martinez, Jacob L. Shelton, Margherita Taddei, Todd D. Krauss, Joseph M. Luther, Md Azimul Haque, Matthew C. Beard

**Affiliations:** † 53405 National Laboratory of the Rockies, Golden, Colorado 80401, United States; ‡ Department of Chemistry, 6927 University of Rochester, Rochester, New York 14627, United States; § RASEI:The Joint CU-Boulder/NLR Energy Institute, Boulder, Colorado 80309, United States

## Abstract

Terminating semiconductor nanocrystals with chiral organic ligands can induce chiroptical properties that combine the chiral character of the ligands with the strong and tunable optical properties of the nanocrystal. However, the synthesis of such chiral-modified nanocrystals is currently limited by solvent incompatibility between polar chiral ligands and nonpolar traditional ligands, as well as by ligand dissolution behavior that ultimately compromises nanocrystal quality and the degree to which chiroptical properties can be manipulated. In this work, we demonstrate a single-step synthesis of chiral cadmium selenide (CdSe) nanoplatelets (NPLs) that eliminates the need for postsynthetic ligand exchange in aqueous solvents, resulting in highly emissive chiral CdSe NPLs. By directly incorporating chiral aminodecanoic acid ligands during synthesis, we achieve in situ ligand binding and chirality transfer to CdSe NPLs. This approach produces CdSe NPLs with high photoluminescence quantum efficiencies of 50% and circular dichroism dissymmetry factors (*g*_CD_) on the order of 10^–4^.

## Graphical abstract

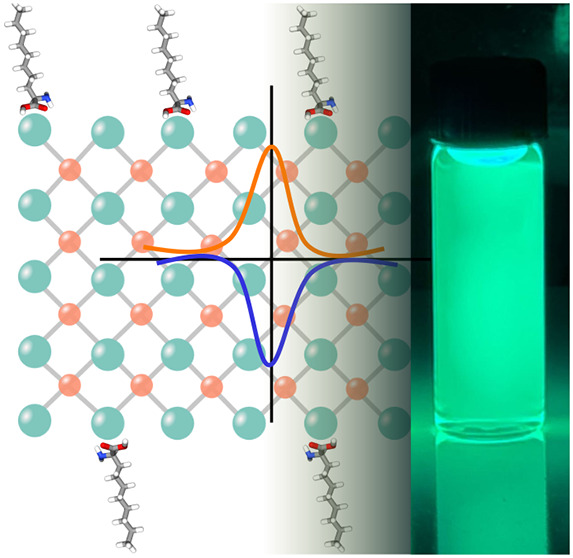

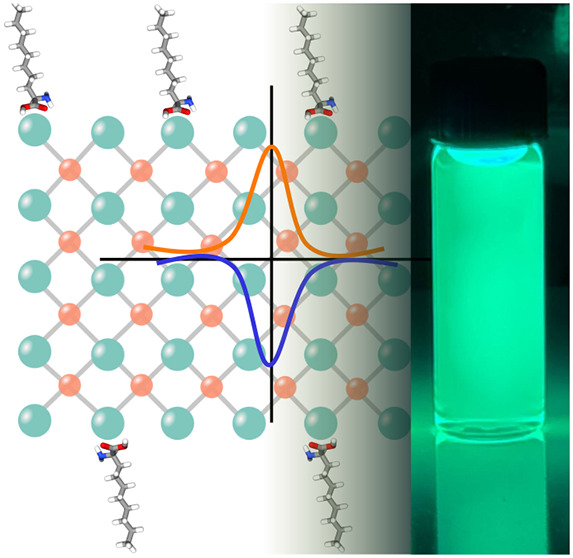

Introducing chirality into semiconductors adds an additional degree of tunability, enabling the absorption and emission of circularly polarized light as well as control over charge-to-spin interconversion, with implications for an array of emerging applications.^1–8^ Chirality in semiconductors can be imparted through several mechanisms, including intrinsically chiral structural defects, formation of chiral heterostructures, and chirality transfer from organic ligands.^9–19^ Among these approaches, ligand-induced chirality is arguably the most experimentally accessible and tunable strategy, and ligand engineering simultaneously plays a critical role in determining the optoelectronic properties and scalability of colloidal semiconductor systems.^20–22^ In colloidal quantum dots and nanoplatelets, postsynthesis ligand exchange with (L/D)-cysteine ligands induces chiroptical properties, as evidenced by circular dichroism (CD) responses.^23^ These chiroptical responses originate from asymmetric surface distortions or hybridization between chiral ligand orbitals and the semiconductor band-edge states.^24^ While postsynthesis exchange of native oleic acid ligands for cysteine ligands is a common approach, it requires a phase transfer of the nanocrystals involving water and the use of highly toxic tetramethylammonium hydroxide.^25^ Ligand exchange circumvents the synthetic challenges associated with maintaining enantiomeric purity and structural uniformity in intrinsically chiral or defect-engineered nanocrystal systems, but it often leads to severe photoluminescence (PL) quenching.^26–28^ This quenching is attributed to surface etching by the strong base and the introduction of nonradiative pathways through thiol-metal coordination at the nanocrystal surface. Since excitonic emission governs the optical performance of semiconductor nanocrystals in light-emitting and photovoltaic applications, preserving PL during chiral modification is essential. Developing synthetic routes that induce chirality without degrading excitonic emission is therefore a critical challenge for advancing chiral semiconductor nanomaterials. Beyond device performance, maintaining bright excitonic emissions also provides a spectroscopic tool for probing chirality transfer mechanisms, offering insights into the interplay between surface chemistry, structural asymmetry, and excitonic dynamics in chiral nanocrystals.

In this study, we investigate colloidal CdSe nanoplatelets (NPLs) owing to their sharp excitonic features and high surface-to-volume ratio. Their ultrathin geometry further enables enhanced sensitivity to surface modifications, making them excellent platforms for examining the effects of ligand incorporation and synthetic variation. We develop a synthetic procedure for achieving in situ chirality transfer from chiral organic ligands to CdSe NPLs while preserving their intrinsic optoelectronic properties. For chiral induction, we explore two chiral ligands, (*R*/*S*)-2-aminodecanoic acid (ADA) and (*R*/*S*)-methylbenzylamine (MBA), introduced alongside native oleic acid ligands during the synthesis of 4.5 monolayer (ML) CdSe NPLs. By incorporating these chiral ligands directly during synthesis, we avoid the challenges typically associated with postsynthetic ligand exchange involving chiral thiols such as cysteine, preserving both the pristine nanocrystal surface and excitonic emission in organic solvents, while simultaneously imparting chiroptical properties to the NPLs. The described synthetic approach provides a simple and effective route for inducing chirality in CdSe NPLs while retaining their narrow and bright excitonic emission, with potential applications in circularly polarized light emission, enantioselective sensing, and advanced chiral photonic devices.

The incorporation of chiral ligands was achieved by modifying the previously reported synthesis for 4.5 ML CdSe nanoplatelets.^29^ The key modification involved the injection of a suspension containing the desired chiral ligand dispersed in oleic acid immediately following the growth of CdSe NPLs, as illustrated in Figure 1a. Oleic acid is one of the most common organic ligands used in semiconductor nanocrystal synthesis, providing steric stabilization and passivating surface trap states.^30,31^ Chiral induction is accomplished by partially replacing these oleic acid ligands with chiral ligands. In the present work, we explore *R*/*S*-ADA and *R*/*S*-MBA as the chiral ligands for chiral induction in CdSe NPLs (Figure 1b). Chiral ligands (0.2 mmol) were introduced alongside oleic acid (6.34 mmol), corresponding to an oleic acid-to-chiral ligand molar ratio of ∼ 32:1. *R*/*S*-ADA was selected based on the hypothesis that its carboxylate functional group would provide strong binding to cadmium-rich facets of the nanocrystal surface, enhancing colloidal stabilization. On the other hand, *R*/*S*-MBA is a widely used chiral amine ligand in halide perovskite nanocrystals and related systems.^32^ Although amine ligands can interact with CdSe NPL surfaces through multiple binding modes,^33^ we assume that both ADA- and MBA-based ligands bind to surface Cd sites (Figure 1c). ADA-derived carboxylates coordinate as X-type ligands to the cadmium-rich top and bottom facets via strong bidentate or bridging interactions with undercoordinated Cd^2+^ sites. In contrast, MBA functions as an L-type ligand, binding more weakly through dative coordination at the same Cd-rich surfaces, with greater lability and a propensity to displace native cadmium-carboxylate species.^34–36^

**Figure 1:**
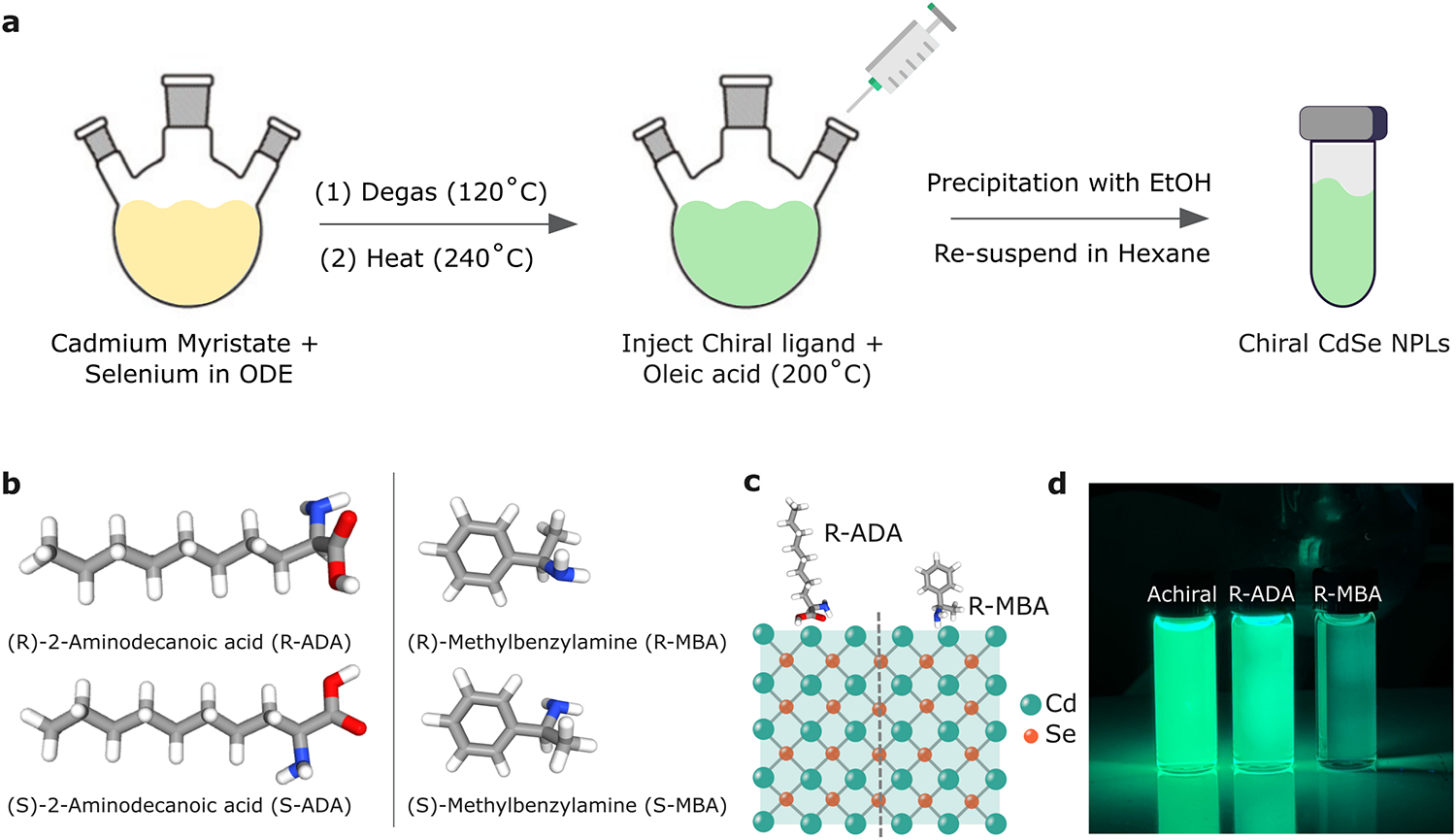
Modified CdSe synthetic procedure for chiral CdSe NPLs. (b) Chemical structure of chiral ligands *R*/*S*-ADA and *R*/*S*-MBA. (c) Schematic representation of the cross-section of a 4.5 ML CdSe NPL with possible binding interactions of ADA- and MBA-based chiral ligands on its surface. (d) Light emission from washed achiral and chiral NPLs under UV light illumination.

Unlike conventional postsynthetic ligand exchange methods that rely on aqueous ligand exchange with chiral thiols such as cysteine which often require phase transfer, the current strategy enables the simultaneous introduction of both achiral and chiral ligands toward the end of the reaction, avoiding surface damage associated with harsher exchange conditions. NPL suspensions in hexane incorporating either ADA or MBA retain their PL emission for several months under ambient conditions, underscoring their excellent colloidal and optical stability. Under UV illumination, ADA-capped NPLs exhibit bright emission, in stark contrast to the markedly weaker emission observed for MBA-capped NPLs (Figure 1d) as discussed later.

Scanning transmission electron microscopy (STEM) imaging of freshly synthesized achiral and chiral CdSe NPL samples reveal the formation of elongated platelet-like morphologies in all cases (Figure 2a) consistent with previous reports.^37,38^ Notably, no twisted assemblies or helical stacking of the CdSe NPLs were observed in STEM imaging, which is consistent with chirality transfer arising at the local surface level rather than from mesoscale structural reorganization.^9,39^ However, we emphasize that these structural observations do not allow us to distinguish between two plausible microscopic origins: (i) asymmetric local lattice distortions induced by chiral ligand binding, or (ii) electronic hybridization between chiral ligand orbitals and the NPL excitonic states at the surface.^9,15,40^ Higher magnification STEM imaging provides further insight into the structural quality of the NPLs as a function of ligand identity (Figure 2b). Achiral oleic acid-capped NPLs exhibit lattice fringes in some regions but not others, suggesting a polycrystalline structure with localized surface disorder. In contrast, chiral NPLs show different degrees of crystalline order depending on the passivating ligand employed. ADA-passivated chiral NPLs appear to possess a partially disordered structure. In contrast, MBA-passivated chiral NPLs exhibit a well-defined single-crystalline structure with minimal surface disorder, suggesting that MBA binds more gently at the nanocrystal surface without significantly perturbing the underlying lattice. This apparent structural preservation in MBA-capped NPLs is somewhat surprising given their inferior PL performance and suggests that surface trap states in this system may arise from chemical rather than structural disorder, consistent with cadmium displacement by the amine ligand rather than lattice disruption. Collectively, these observations highlight the critical and ligand-specific role of surface passivation in determining the crystalline quality, surface order, and ultimately the optical and chiroptical properties of chiral CdSe NPLs.

**Figure 2:**
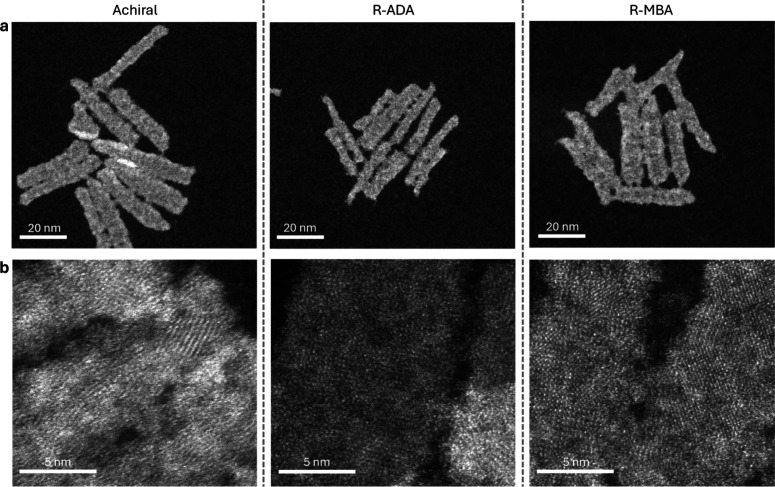
STEM images of achiral and chiral CdSe NPLs passivated with oleic acid and ADA or MBA ligands at (a) lower and (b) higher magnifications.

The absorption spectra of CdSe NPLs capped with ADA and MBA ligands (Figure 3a,b) exhibit two distinct sharp excitonic peaks corresponding to heavy- and light-hole excitonic peaks characteristic of 4.5 ML CdSe NPLs.^41,42^ The narrow peaks reflect the uniformity at the single monolayer-level in the platelet thickness. This is in contrast to postsynthetic approaches, where excitonic peaks typically broaden following ligand exchange, possibly due to changes in the ligand density and ripening effects.^13^ Consistent with the absorption spectra, sharp PL emission is observed for both ADA and MBA-capped CdSe NPLs (Figure 3c,d, Figure S1). Notably, MBA-capped CdSe NPLs exhibit marginally narrower excitonic absorption and PL peaks compared to ADA-capped CdSe NPLs, though a weak absorption and emission feature at 460 nm indicates the presence of a small amount of 3.5 ML NPLs formed during synthesis.^43^ Relative PL quantum yield (PLQY) measurements reveal a striking difference between the two ligand systems. ADA-capped CdSe NPLs exhibit a PLQY of ∼ 50% (Figure 3e), exceeding that of pristine oleate-capped NPLs (∼34%),^44^ indicating more effective surface passivation. We attribute this to the steric compatibility between oleic acid and ADA as coligands, as well as the strong and directional binding of ADA to the cadmium-rich nanocrystal surface through its carboxylate group. In contrast, MBA-capped CdSe NPLs exhibit a PLQY nearly 1 order of magnitude lower (Figure 3f), consistent with their weaker emission observed under UV illumination (Figure 1c). The PL quenching observed in MBA-capped NPLs is likely multifactorial. Primary amines such as MBA bind more weakly to nanocrystal surfaces than carboxylate ligands, and their dynamic binding behavior can expose undercoordinated surface sites.^33,35,45^ Additionally, amines can actively displace native cadmium-carboxylate surface species, effectively stripping cadmium from the NPL surface.^34^ This loss of surface cadmium creates anion-rich surface sites that can act as nonradiative recombination centers, resulting in significant PL quenching.^42^ The concurrent appearance of a 3.5 ML secondary CdSe phase in MBA-capped NPLs further supports amine-driven disruption of surface cadmium stoichiometry. The interplay between weak amine binding, cadmium displacement, and the resulting increase in surface trap density collectively account for the inferior emission of MBA-capped NPLs relative to ADA-capped NPLs. Overall, the in situ ligand strategy employed here effectively preserves the intrinsic excitonic absorption and emission properties of the CdSe NPLs, yielding superior optoelectronic characteristics compared to postsynthetic ligand exchange routes.

**Figure 3:**
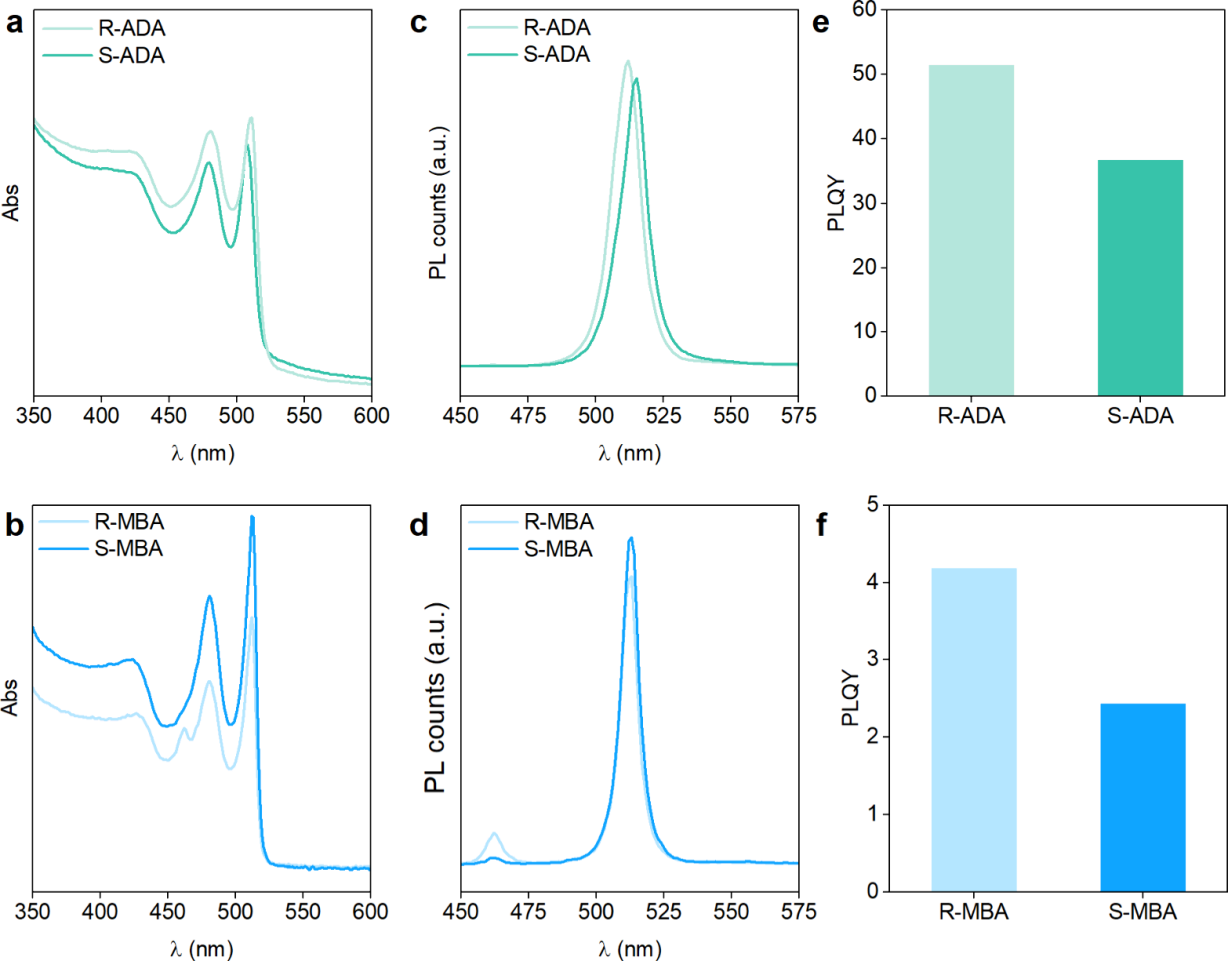
Optical properties of ADA and MBA-capped CdSe NPLs. (a,b) Absorbance spectra. (c,d) PL spectra. (e,f) PLQY.

Circular dichroism (CD) spectroscopy, which measures the differential absorption of left- and right-handed circularly polarized light, provides direct evidence of chiroptical activity and chirality transfer. Both ADA- and MBA-capped CdSe NPLs exhibit well-defined CD responses with onset at the band edge, consistent with their respective absorption spectra and confirming successful chirality transfer from the chiral ligands to the CdSe NPL host. As expected, achiral oleic acid-capped CdSe NPLs show no CD signal, consistent with their centrosymmetric nature (Figure S2). The CD spectra of ADA-passivated NPLs show polarized light absorption feature arising at the heavy-hole exciton transition at approximately 512 nm, with a weaker feature at the light-hole transition (Figure 4a). *R*-ADA and *S*-ADA enantiomers produce CD signals of opposite sign and comparable magnitude, yielding mirror-image CD spectra confirming the successful chirality transfer to CdSe NPLs from the chiral ligands. Similarly, MBA-passivated NPLs exhibit mirror-image CD responses for the R and S enantiomers, with CD features again coinciding with the excitonic transitions (Figure 4b), further confirming the generality of the in situ chirality transfer approach across different ligand chemistries. The dissymmetry factors (*g*_CD_) for both ADA- and MBA-capped CdSe NPLs are on the order of 10^–4^ (Figure 4c,d), comparable to those reported for chiral CdSe NPLs prepared via postsynthetic ligand exchange. Room-temperature circularly polarized luminescence (CPL) measurements on *R*/*S*-ADA-capped CdSe NPLs did not yield a significant difference in left- and right-handed circularly polarized emission, consistent with the known suppression of CPL signals at room temperature due to thermal broadening and depolarization effects. Low-temperature CPL measurements may reveal more pronounced chiral emission dissymmetry and provide deeper insights into the chiroptical properties of these systems.

**Figure 4:**
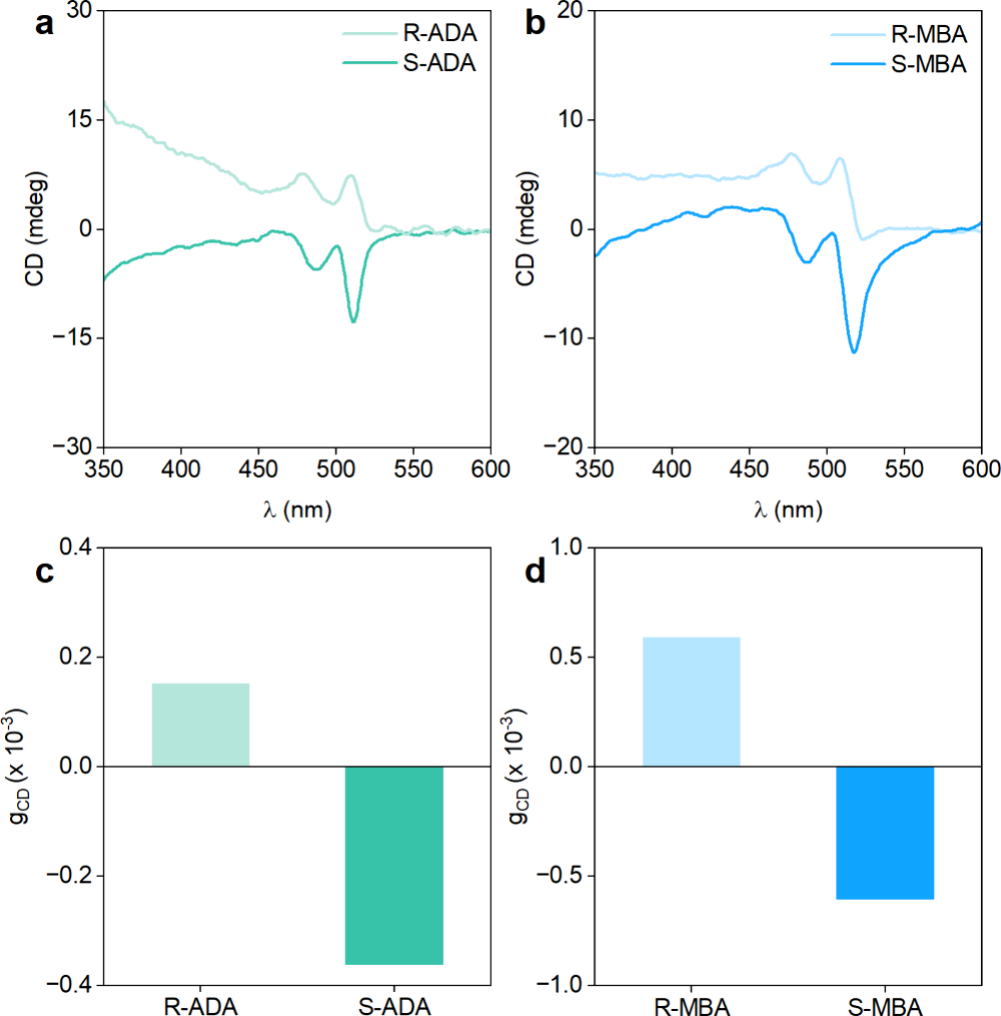
Chiroptical properties of ADA and MBA-capped CdSe NPLs. (a,b) Circular dichroism spectra. (c,d) *g*_CD_ values calculated at the exciton absorption peak at 511 and 512 nm for ADA and MBA capped CdSe NPLs, respectively.

This study demonstrates a single-step synthetic approach for chiral CdSe NPLs, utilizing in situ incorporation of *R*/*S*-ADA or *R*/*S*-MBA chiral ligands during NPL synthesis. While dissymmetry factors are comparable to those achieved by postsynthetic ligand exchange, this approach preserves the intrinsic excitonic properties and optoelectronic quality of the NPLs. ADA-capped NPLs exhibit high PLQY alongside sharp excitonic absorption and emission features, confirming that nanoplatelet surface integrity and the quantum-confined electronic structure are maintained upon chiral ligand incorporation. CD measurements confirm successful chirality transfer from the molecular ligands to the CdSe NPLs, with *g*_CD_ values on the order of 10^–4^, and mirror-image CD responses for R and S enantiomers validating stereochemical chirality transfer. Collectively, this work highlights the importance of ligand selection and in situ ligand engineering in tailoring the chiroptical properties of colloidal CdSe.

## Supplementary Material



## Notes

Additional absorption and photoluminescence data, relative quantum yield details, circular dichroism of achiral sample (PDF)
