# Supplementary material for: In-Situ Ligand-Induced Chirality Transfer in Emissive CdSe Nanoplatelets
Source: J Phys Chem Lett. 2026 Jun 17;17(26):7261–6. doi: 10.1021/acs.jpclett.6c01325 (PMC13339767; doi:10.1021/acs.jpclett.6c01325)
Supplement: Supplementary file 1 [file jz6c01325_si_001.pdf]

Supporting Information

In-situ Ligand-Induced Chirality Transfer in Emissive CdSe Nanoplatelets

William Girten,[1,2] Farwa Awan,[2] Nikita S. Dutta[1] Marissa Martinez,[1] Jacob L. Shelton,[1] Margherita Taddei,[1] Todd D. Krauss,[2] Joseph M. Luther,[1,3] Md Azimul Haque,[1,3]* Matthew C. Beard[1,3]*

[1]National Laboratory of the Rockies, Golden, CO, 80401, USA
[2]Department of Chemistry, University of Rochester, Rochester, NY, 14627, USA
[3]RASEI:The Joint CU-Boulder/NLR Energy Institute, Boulder, CO, 80309, USA

mdazimul.haque@nlr.gov, matt.beard@nlr.gov

EXPERIMENTAL METHODS

Chemicals. Cadmium acetate dihydrate ($Cd(OAc)_2 2H_2O$) (98%), sodium myristate (≥99%), selenium powder (≥99.5%), technical grade 1-octadecene (ODE), technical grade oleic acid (OA), and 3-methylpentane (≥99%) were purchased from Sigma-Aldrich. Hexane and methanol were purchased from Fisher Chemical. R/S-α-methylbenzylamine (R/S-MBA) and R/S 2-Aminodecanoic acid (R/S-ADA) were purchased from Sigma-Aldrich and Ambeed Inc., respectively.

Preparation of Cadmium Myristate: Cadmium nitrate tetrahydrate (3g) was first dissolved in 200 mL methanol and in a separate beaker, sodium myristate (5g) was dissolved in 500mL of methanol. The solution containing the cadmium nitrate tetrahydrate was then added dropwise to the sodium myristate and stirred for 2 hours. The resulting suspension was vacuum filtered and washed with excess methanol. The resultant powder was dried under vacuum overnight and stored in a glovebox for future use.

Synthesis of 4.5 ML CdSe NPLs: In a typical synthesis, 180 mg of dried cadmium myristate and 30 mg of selenium powder were combined in 15 mL of ODE in a 100 mL three neck round bottom flask. The solution was then degassed under vacuum at 120 ^{o}C for 1 hour. The mixture was then put under nitrogen flow, and the temperature was raised to 240 ^{o}C. When the temperature reached 210 ^{o}C, cadmium acetate dihydrate (0.3-0.1 mmol) was added swiftly into the reaction mixture. The temperature of the solution was then kept at 240 ^{o}C for 8 minutes and then placed in a water bath. When the temperature reached 190 ^{o}C, 2 mL of OA was rapidly injected. Once the mixture reached room temperature, 15 mL of hexane was injected, and the solution was centrifuged at 3000 rpm for 10 minutes. The supernatant was discarded and the pellet containing the NPLs was redispersed in 12 mL of hexane and allowed to sit for at least one hour before centrifugation at 6000 rpm for 10 minutes. The supernatant containing the NPLs was kept and stored in the dark under ambient conditions for further use.

Synthesis and purification of Chiral CdSe NPLs: Incorporation of the chiral ADA and MBA ligands is carried out in the final stages of the NPL synthesis above. Following the cooling of the solution to 190 ^{o}C, a suspension of either ADA (0.2 mmol) or MBA (0.2 mmol) in 2 ml of oleic acid was swiftly injected. The suspension was sonicated for 30 minutes prior to preparation of the materials for the synthesis. Following the synthesis, the chiral NPLs were precipitated several times using ethanol and centrifuged at 6000 rpm for 10 minutes, re-suspending in hexanes.

Scanning Transmission Electron Microscopy: Scanning transmission electron microscopy (STEM) data was acquired on a Thermo Fisher Scientific Spectra 200 S/TEM equipped with a probe Cs corrector. Samples were prepared by drop casting on lacey carbon grids with ultrathin carbon supports (Ted Pella). Grids were plasma cleaned at 25W for 45s using N_2 gas with 5 mol% H_2 before loading into the S/TEM. Imaging was performed at 200kV in high-angle annular dark field (HAADF) STEM mode with a convergence angle of 24.2 mrad.

UV-VIS, CD, and Fluorescence Spectroscopic Measurements: UV-VIS absorption spectroscopy was performed on a PerkinElmer Lambda 950 UV/VIS spectrophotometer. CD measurements were carried out using Olis DSM 170 spectropolarimeter. PL measurements were collected using an in-house-built fluorometer setup with a 450 W Xenon arc lamp source, which is coupled to an excitation SpectraPro 150 monochromator. A photomultiplier tube (PMT) PL coupled with an emission SpectraPro 300i monochromator was used for PL detection. All sample measurements were collected in a 1cm path length Infrasil cuvette. All PL measurements were corrected for baseline and detector efficiency correction.

Relative Quantum Yield: QY measurements were calculated relative to Coumarin 153/540A dye acquired from Luxottica Exciton. The coumarin dye solution was prepared in ethanol, and the reference QY for the dye is reported at 0.53 by the manufacturer.

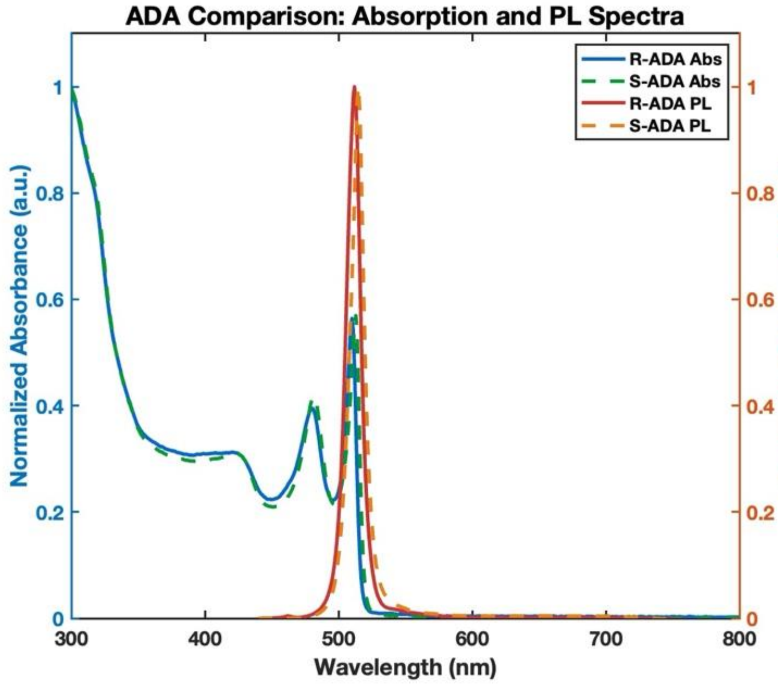

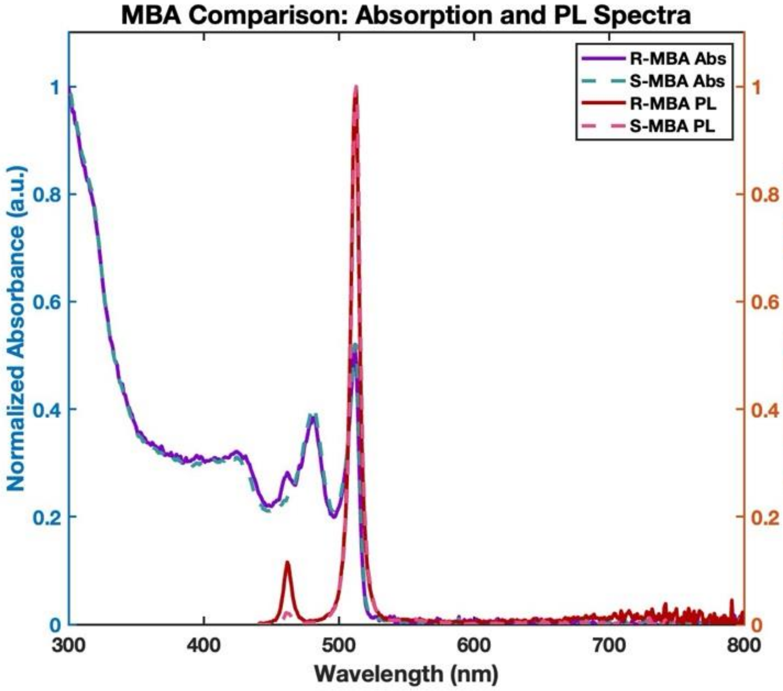

Figure S1. Absorption and PL spectra for ADA and MBA-capped CdSe NPLs.

Relative Quantum Yield

Dye: Coumarin 153/540A
Excitation λ = 430nm
Refractive index of hexanes: 1.375
Refractive index of ethanol: 1.361
Absolute QY of Dye as per manufacturer: 0.53 or 53%

Sample	Relative QY %	Integrated PL	Absorbance at 430nm	Solvent
**Coumarin 153/540 A**	-	1.445e+07	0.07	Ethanol
**R-ADA**	51.45	2.3166e+07	0.11852	Hexanes
**S-ADA**	36.73	2.5563e+07	0.18318	Hexanes
**R-MBA**	4.18	7.6988e+05	0.048495	Hexanes
**S-MBA**	2.43	6.2928e+05	0.068141	Hexanes

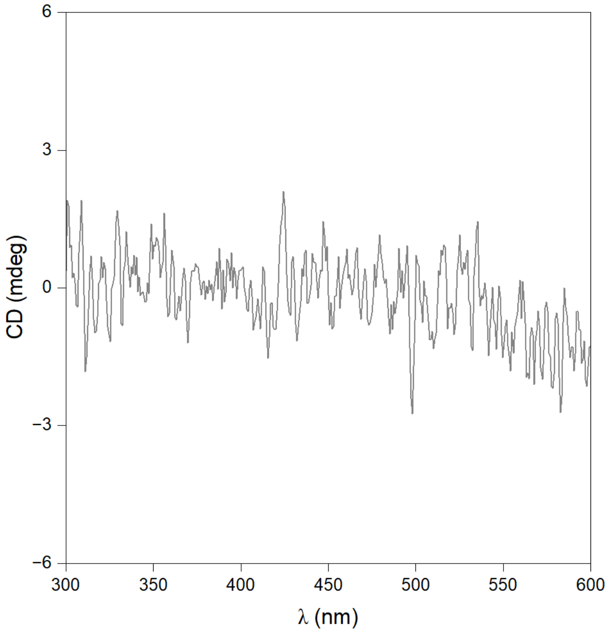

Figure S2. CD spectrum of achiral CdSe NPLs.
